# Long-Duration Vocational Education’s Effects on Individuals’ Vocational Identity, Self-Efficacy, and Job Satisfaction

**DOI:** 10.3390/bs15091161

**Published:** 2025-08-26

**Authors:** Cailing Yan

**Affiliations:** The Institute of Vocational Education, Tongji University, Shanghai 200092, China; yaneva2021@tongji.edu.cn

**Keywords:** long-duration vocational education (LDVE), vertically integrated vocational education (VIVE), vocational identity, self-efficacy, job satisfaction, PSM

## Abstract

This paper focuses on students receiving vertically integrated vocational education and evaluates the impacts of long-duration vocational education (LDVE) on individuals’ vocational identity, self-efficacy, and job satisfaction. Based on 1878 survey data, a logit model was used to analyze the influencing factors of students’ participation in the vertically integrated vocational education (VIVE) program, and the propensity score matching (PSM) method was used to measure the effects. Moreover, the comparison was made in different VIVE models to find the heterogeneity of effects. The results showed that family background and major significantly negatively affected the odds of an individual receiving VIVE. Merely extending the duration of vocational education does not significantly improve vocational identity, self-efficacy, and job satisfaction. Furthermore, the results of the between-group difference analysis indicated that neither the secondary-to-higher VIVE model nor the higher-to-undergraduate VIVE model had a significant impact on individuals’ vocational identity, self-efficacy, and job satisfaction. This research finding expands on the research achievements regarding the effects of LDVE. It can provide data references for the government and relevant institutions to pay attention to the quality and potential influence factors of LDVE.

## 1. Introduction

Vocational education plays an important role in modern society. For example, over the past decade, China’s vocational education has cumulatively delivered 61 million high-quality laborers and technically skilled personnel to all walks of life, and more than 70 percent of the new frontline employees in modern manufacturing, strategic emerging industries, and modern service industries are graduates from vocational education (MoE, 2023). Vocational education has been seen as an important tool in the development of the economy, and it has numerous examples of presumed effects in different countries, regions, and specific sectors (Sims, 2004; Budría & Telhado-Pereira, 2009). Vocational education equips students with practical skills and professional knowledge, facilitating employment and improving long-term job stability. Some data show that the stable employment rate of vocational education graduates exceeds that of general education graduates before the age of 40 (Woessmann, 2019).

In addition to imparting job skills and professional knowledge, vocational education significantly influences students’ vocational perceptions and the internalization of professional roles (Skorikov & Vondracek, 2011). Research has shown that vocational education can significantly impact students’ vocational identity, self-efficacy, and job satisfaction by offering them internships and career guidance (Billett, 2011). Career development theories further emphasize that vocational identity, self-efficacy, and job satisfaction are critical determinants of occupational longevity. For instance, the life-span theory of career development (Super, 1980) proposes that individuals with a strong vocational identity tend to perceive their careers as integral to their self-concept, thereby fostering long-term commitment. Similarly, Social Cognitive Career Theory (Lent et al., 2006) identifies self-efficacy as a pivotal antecedent of occupational behaviors, with higher self-efficacy correlating with more adaptive and sustained career engagement. Empirical support also derives from Herzberg’s Two-Factor Theory (Herzberg et al., 2011), which distinguishes between hygiene factors and motivators, positing that satisfaction derived from motivators significantly enhances occupational stability.

On these bases, China’s vocational education system offers vertically integrated vocational education (VIVE) programs, representing a specific LDVE model in China. It requires students to complete years of vocational education after graduating from junior school or senior high school to obtain an associate’s or bachelor’s degree. According to the international standard classification of education, VIVE programs lie between short post-secondary programs at ISCED 5 and long first-degree programs at ISCED 6. There are three types of VIVE: the secondary-to-higher vocational education model, the higher-to-undergraduate vocational education model, and the secondary-to-undergraduate vocational education model. The secondary-to-higher vocational education model requires students to determine their vocational education path by the end of secondary school and obtain an associate degree over the subsequent five years. The higher-to-undergraduate vocational education model requires students to complete their vocational education continuously, from higher vocational education to undergraduate vocational education, to obtain a bachelor’s degree. The “Action Plan for Improving the Quality and Excellence of Vocational Education (2020–2023)” in China stipulates the gradual elimination of the secondary-to-undergraduate vocational education model to standardize the sustainable cultivation of technical and skilled talents while moderately expanding the other two integration models. Thus, in China, the secondary-to-higher vocational education and higher-to-undergraduate vocational education models are the two main ways individuals receive LDVE.

Extending the duration of vocational education aims to enhance vocational college students’ vocational identity, self-efficacy, and job satisfaction. This approach seeks to cultivate more potential skilled talents to address the shortage of technical professionals. According to social learning theory, career decision-making is shaped by learning experiences (Mitchell, 1974). Vocational education experiences may, therefore, encourage more people to choose careers in skilled labor. However, does this imply that extended long-term vocational education has a significant effect? Furthermore, does LDVE lead to a better vocational identity, self-efficacy, and job satisfaction? Limited research addresses such topics. This paper aims to investigate the impact of LDVE on individuals’ vocational identity, self-efficacy, and job satisfaction by analyzing data from participants in the VIVE programs. Such research can not only help expand the research on vocational education but also offer more empirical evidence for optimizing vocational education practice.

## 2. Literature Review

### 2.1. Study of the Effects of Vocational Education

Many studies have demonstrated a significant positive correlation between years of schooling and personal income, business earnings, and other related factors (Angrist & Krueger, 1991; Bils & Klenow, 2000; Patrinos & Psacharopoulos, 2020). The most renowned is the Mincer Equation, formulated by Milton Friedman and Jacob Mincer. The equation suggests that each additional year of education typically results in a significant increase in an individual’s income level. Human capital theory (Becker, 1967) posits that prolonged engagement in education enhances human capital accumulation, thereby generating measurable economic returns. Research on the marginal effects of vocational education has also confirmed that individuals who complete a full vocational education have higher employment rates, earnings, and employment stability than those who do not (Ryan, 2002a, 2002b).

Furthermore, with the rapid advancement of technology, the cycle of occupational transitions will become increasingly shorter. In this context, a study has demonstrated that vocational skills training with relevant supplementary qualification content is of great significance, which would be the main approach to coping with the shortage of skilled workers in the next fifteen years (Hall et al., 2016). However, there are also studies pointing out that in some cases, overeducation or skill mismatch may lead to negative marginal benefits, such as lower job satisfaction and higher turnover. (Battu et al., 2000; Krueger & Lindahl, 2001).

### 2.2. Vocational Education and Vocational Identity

Vocational identity refers to an individual’s self-awareness and acceptance of their occupational role, reflecting their recognition of the job and commitment to the career (Super, 1980; J. Holland et al., 1980; Skorikov & Vondracek, 2011; Porfeli & Lee, 2012). It encompasses the recognition of the requisite knowledge and skills for the position, as well as the internalization of the social values and responsibilities inherent in the profession. Career stability, job satisfaction, career commitment, and professional achievement have been significantly impacted by vocational identity (Skorikov & Vondracek, 2011; Hirschi, 2012).

Research has revealed that vocational education, but not long-duration vocational education, can positively influence an individual’s vocational identity through the acquisition of skills and practical experience in a broad sense (Savickas, 2013). Some scholars have noted that students who participate in real-world work projects or internships are more likely to develop a sense of identity, as they learn to assume duties and responsibilities in a real work environment (Dewey, 1938; J. L. Holland, 1973). Except for the schooling time, vocational education also has a sustained effect on their vocational identity early in their careers (Kunnen, 2013). In summary, vocational education can help students adapt to their occupational roles more quickly after leaving school and further strengthen their vocational identity as they gain experience in their careers (Savickas, 2013).

### 2.3. Vocational Education and Self-Efficacy

Self-efficacy refers to an individual’s belief in their ability to perform a task successfully (Bandura, 1997). Individuals with higher self-efficacy are more likely to positively identify with their jobs because they are more confident in their work and believe that they can cope with challenges at work successfully (Betz & Hackett, 2006; Choi et al., 2012). In addition, self-efficacy can increase an individual’s sense of mastery over occupational tasks, which can further enhance their identification with their job.

Self-efficacy is influenced by four principal sources of information: performance accomplishments, vicarious experience, verbal persuasion, and physiological states (Bandura, 1997). One of the features of vocational education is the integration of theory and practice. Enhancing self-efficacy through direct performance is a fundamental advantage of vocational education. Research has shown that students who participate in hands-on exercises and internships tend to increase their self-efficacy through direct experience (Oje et al., 2021). Students could build up their confidence in task completion and cultivate trust in their abilities. In addition, vocational education often employs simulations, case studies, and task-based learning as teaching methods, enabling students to experience and reflect in a genuine and safe environment while gaining access to important theories and practical skills. This ‘vicarious experience’ enables students to realize that they can undertake similar tasks, thus increasing their self-efficacy.

Furthermore, teachers in vocational education play a crucial role in shaping students’ self-efficacy. In vocational education, the teacher-student relationship is more diverse than in general education, where the vocational teacher is not only a knowledge transmitter but also a technical mentor and career development advisor. Research has found that positive feedback and support, including teacher verbal persuasion, can significantly increase students’ self-efficacy (Zeldin et al., 2008; Turda et al., 2021). In particular, when students encounter difficulty, teachers’ verbal encouragement and guidance may help them develop psychological resilience and enhance their confidence in facing career challenges. In addition, teachers can build positive physiological states in students by facilitating successful learning experiences through the provision of moderately challenging tasks and delivering affirmative feedback upon task completion, thereby enhancing their self-efficacy (Pekrun, 2006).

### 2.4. Vocational Education and Job Satisfaction

Job satisfaction is a multidimensional concept referring to an individual’s overall affective evaluation of their job. “Job satisfaction is a pleasurable or positive emotional state resulting from the appraisal of one’s job or job experiences” (Locke, 1976). Many studies underscore the significance of job satisfaction, which could impact employee performance, productivity, turnover intentions, and overall well-being (Judge et al., 2001; Faragher et al., 2005).

Studies have confirmed that job fit, defined as the match between an individual’s qualifications and job requirements, significantly determines job satisfaction (Cable & Judge, 1996; Rauner & Maclean, 2008). Vocational education graduates tend to have a higher level of job satisfaction than general education graduates because their skills align more closely with the job requirements, thereby improving job satisfaction (Vila & García-Mora, 2005). Winkelmann (1996) analyzed the positive impact of German apprenticeships on the job satisfaction of vocational education graduates, affirming that skills obtained through vocational education align more closely with job requirements, thereby enhancing employee job satisfaction. The application of job analysis approaches in vocational education to acquire job information, which helps the development of training programs, evidently facilitates the alignment of skills with employment requirements, enhancing job satisfaction (Schneider & Konz, 1989).

## 3. Research Hypotheses

Generally, most previous studies have been conducted at the macro level, focusing on the marginal effects of education. Empirical studies corroborate this proposition, demonstrating that extended educational attainment significantly reduces the risk of unemployment (OECD, 2021). Building on this framework, the learning curve theory (Argote & Epple, 1990) further clarifies how sustained vocational training accelerates the mastery of competency, leading to quantifiable economic benefits. For instance, research on higher education in medical science reveals a positive association between years of clinical training and diagnostic accuracy (Ericsson et al., 1993). However, despite these insights, limited empirical attention has been paid to vocational education, specifically. Given that vocational education constitutes a distinct form of human capital investment, it seems plausible to hypothesize that LDVE may yield similar positive marginal effects. However, in China, although it has established the largest vocational education system in the world, there still exists a severe supply-demand imbalance of skilled workers. The job-to-applicant ratio for technical workers in China has remained above 1.5, while the job-to-applicant ratio for senior technicians exceeds 2 (Li, 2021).

Additionally, vocational education was often stereotyped as unprestigious, and its students as stupid and disobedient (Van de Weerd, 2022). It is usually only students with poor academic performance who attend vocational schools. According to a study of 21,531 junior high school students in China, poor grades were the primary reason for attending secondary vocational education (Y. L. Shen, 2022). Such stereotypes about vocational education and its students are common in China (Bao et al., 2023); they may influence the educational contribution of vocational education.

Although some existing studies have confirmed the positive effects of vocational education on vocational identity, self-efficacy, and job satisfaction, there is a lack of research examining the influence of LDVE on these variables. In fact, VIVE, as a representative LDVE program, has been established for years. However, the shortage of skilled workers and negative stereotypes of vocational education in China are also long-standing problems, which question the positive effects of vocational education. Thus, this study analyzed whether there exist significant effects of LDVE on vocational identity, self-efficacy, and job satisfaction. Consequently, three research hypotheses are proposed.

**Hypothesis 1:**  *LDVE significantly affects individuals’ vocational identity (H1).*

**Hypothesis 2:**  *LDVE significantly affects individuals’ self-efficacy (H2).*

**Hypothesis 3:**  *LDVE significantly affects individuals’ job satisfaction (H3).*

## 4. Research Method

### 4.1. Measurement Model

To assess the effect of LDVE on individuals’ vocational identity, self-efficacy, and job satisfaction, this study has constructed a measurement model with the following expression:

Y_i_ = α + δD_i_ + βX_i_ + ε (1)

In Equation (1), Y_i_ indicates the degree of the outcome variable. D_i_ indicates LDVE experience. In this research, whether students participate in the VIVE programs is the variable referring to the LDVE experience. Meanwhile, δ represents the degree of influence of VIVE on individuals’ vocational identity, self-efficacy, and job satisfaction, respectively. X_i_ refers to other control variables, and β is the coefficient of these control variables. Additionally, α is the constant term, and ε is the interference term.

If individuals randomly choose whether to receive VIVE, δ in Equation (1) represents the net effect of LDVE. However, whether to receive LDVE, like participating in a VIVE program, is not randomly determined. Factors such as personal characteristics, family background, age, and educational content can all influence educational selection. For example, many studies have proven that gender, as a typical personal characteristic, can significantly affect participation, interaction, and interest in education (Eccles, 1994; Perna, 2004). Being from rural or urban areas often implies different family economic status, family structures, and parental education and support, all of which can influence educational development (Davis-Kean, 2005; Jeynes, 2007). The chosen major or subject, which determines the learning content, such as curricula, entails different requirements and competition and could influence educational decisions (Xu, 2013).

Previous studies have treated the acceptance of vocational education as a variable without controlling for confounding factors, which can lead to sample selection bias in the estimation results. The PSM method is commonly employed to address this problem. Based on similar propensities, individuals who receive VIVE and those who do not are matched to ensure the two groups are balanced and comparable. Their degree of vocational identity, self-efficacy, and job satisfaction can then be compared. Research has shown that PSM, as a classic method of quasi-experimental design rooted in the counterfactual framework and the establishment of a control group, can well mimic the randomized assignment to treatment and comparison groups to solve the problem of selection bias, thus obtaining more accurate analysis results (Wooldridge, 2002). The odds of individuals receiving VIVE under given conditions are obtained by the method of Dehejia and Wahba (2002), who employed the logit model to calculate the propensity matching score. The specific expression is listed below:

P (Z_i_) = P (D_i_ = 1│Z_i_) = (exp (Z_i_ ‘β))/(1 + exp (Z_i_ ‘β)) (2)

In Equation (2), P(D_i_ = 1│Z_i_) is the propensity matching score or odds of an individual receiving VIVE, and Zi is the matching variable. In this research, the matching variable includes gender, family background, and major. After obtaining the propensity matching score, an appropriate matching method is selected to match the group that does not receive VIVE. Then, the average treatment effect on the treated (ATT) is used to estimate the effect of LDVE on individuals’ vocational identity, self-efficacy, and job satisfaction, respectively. The expression is as follows:

ATT = E(Y_1_│D = 1) − E(Y_0_│D = 1) = E(Y_1_ − Y_0_│D = 1) (3)

To assess the statistical significance of this effect, independent samples *t*-tests are applied to compare the mean outcomes between the matched treatment and control groups. Although this procedure estimates group differences in a manner similar to other quasi-experimental approaches, such as Difference-in-Differences (DID), this study does not employ DID because no time-series (pre/post) data is available. The estimation relies entirely on cross-sectional matching based on observed covariates.

In Equation (3), Y_1_ denotes the outcome variable, including vocational identity, self-efficacy, and job satisfaction of the group that receives VIVE; Y_0_ represents, respectively, the vocational identity, self-efficacy, and job satisfaction of the group not receiving such education.

### 4.2. Sample Collection

This study adopted convenience sampling and distributed online questionnaires to vocational students who have participated in VIVE in seven cities in China. The researcher initially contacted the vocational institutes via personal connections and the researcher’s workplace platform, and it was extended by snowball sampling. Subsequently, a thorough online webinar was conducted to obtain consent from participants. Potential participants were explicitly informed that their participation in the survey was voluntary and that they could leave at any time without negative consequences. After deleting invalid questionnaires caused by an unusually short time and random responses, such as identical answers for all questions, a total of 1878 questionnaires were deemed valid and saved.

The respondent pool consisted of 46.2% males and 53.8% females. Geographically, rural respondents formed the majority, representing 59.5%, while urban respondents made up 40.5%. Generally, rural areas in China have a resident population of fewer than 3000, whereas urban areas have a resident population of more than 3000. This distribution aligned well with the actual distribution of students’ family backgrounds and the fact that most vocational education students in China originate from rural regions (MoE, 2023). Notably, the number of secondary vocational schools in China has decreased in recent years, indicating a trend of reduced enrollment. Additionally, most vocational universities established after 2019 have a relatively smaller student body. Therefore, the respondents from vocational colleges were the majority. The distribution of majors among respondents predominantly included engineering and manufacturing, business, medical and nursing, and information technology, with over 50% of students originating from engineering and manufacturing and business disciplines. This distribution of majors is consistent with the actual situation of vocational education programs in China. Given that vocational education in China is primarily oriented toward employment, the programs offered focus mainly on technical fields, business, and other related disciplines (Table 1).

### 4.3. Variable’s Measurement

This study divided the research variables into dependent, independent, and control variables. The dependent variables included vocational identity, self-efficacy, and job satisfaction. Vocational identity was measured using existing items from the Organizational Identification Scale (Mael & Ashforth, 1992), the Professional Commitment Scale (Suddaby et al., 2009), and the Job Engagement Scale (Rich et al., 2010). In this research, seven items were formed to measure vocational identity. Self-efficacy was measured using the six items from the Task Self-Efficacy Scale (Lucas et al., 1997). Job satisfaction was measured using five items from the Work Experience Questionnaire (Luk & Chan, 2020) and the Internship Satisfaction Questionnaire (Hussien & La, 2018). These questions were developed using a 5-point Likert scale, which ranged from “totally disagree” (1 point) to “strongly agree” (5 points).

The Cronbach’s α values for vocational identity, self-efficacy, and job satisfaction were 0.962, 0.965, and 0.969, respectively, all of which comfortably exceeded the minimum recommended threshold of 0.7 (Tavakol & Dennick, 2011). These results suggested that the questionnaire demonstrated commendable reliability. The KMO value for the questionnaire was 0.768, which was above the acceptable threshold of 0.6 (Kaiser, 1974). Furthermore, Bartlett’s test of sphericity was significant (*p* < 0.001), indicating that the questionnaire demonstrated favorable validity.

The independent variable is receiving LDVE, with 1 indicating participation in VIVE programs and 0 representing non-participation. Considering the existing research on individual, family, and learning factors that affect educational participation, the control variables in this research mainly include gender, family background, and major. In this context, 1 denotes male, while 2 indicates female for gender classification. Regarding family background, 1 represents urban, while 2 signifies rural. Regarding the majors, 1, 2, 3, and 4 correspond to engineering and manufacturing, business, medicine and nursing, and IT, respectively.

## 5. Results

### 5.1. Estimation Before Matching

To examine differences between the treatment group (participants who received LDVE, n = 325) and the control group (those who did not, n = 1553), independent samples *t*-tests were conducted on the key outcome variables: vocational identity, self-efficacy, and job satisfaction. The results showed no statistically significant difference in these outcome variables between the two groups prior to matching (see Table 2). These findings suggest that, before matching, the groups were already relatively similar in terms of vocational identity, self-efficacy, and job satisfaction.

However, it should be noted that simple *t*-tests may be subject to selection bias, as they do not account for pre-existing differences in observed or unobserved covariates between the groups. Therefore, these results should be interpreted with caution and are used here only to provide a preliminary check before applying PSM, which more rigorously adjusts for covariate imbalance.

### 5.2. Propensity Score Estimation

The logit model was initially employed to analyze the odds of individuals receiving VIVE (see Table 3). The results of the logit model showed that no matter the dependent variable, vocational identity, self-efficacy, or job satisfaction, family background and major had significant negative effects on the probability of individuals receiving VIVE.

### 5.3. Propensity Score Matching Analysis

The quality of matching would directly affect the final estimation results. Ideal matching should satisfy both the common support condition and the balancing hypothesis. Typically, the common support condition is met when each treated unit has a comparable control unit with a similar propensity score, whereas the balancing hypothesis requires that the characteristics of the matched treated and control groups are balanced, with no significant differences (Qiu, 2020).

**Figure 1:**
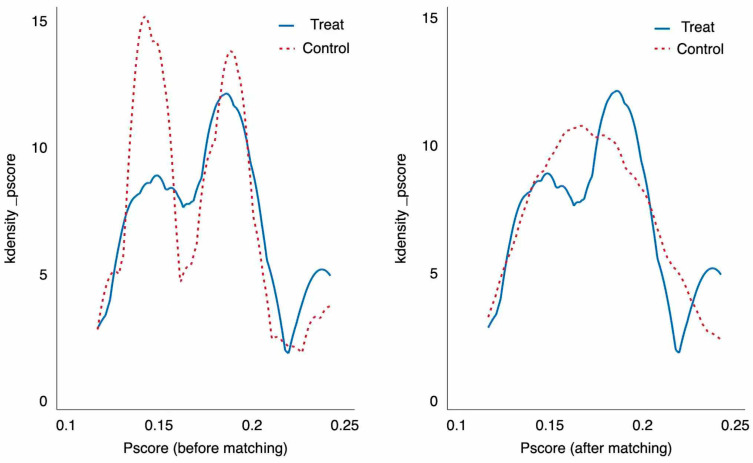
Kernel density estimation before and after PSM.

Figure 1 illustrates the distribution of the predicted propensity scores for individuals participating in VIVE and those who do not. As shown in Figure 1, the propensity scores of the two groups differed more significantly before matching; however, the distributions of propensity scores largely resided within a substantial common support zone after matching, indicating a considerable reduction in the gap between the groups and the effective fulfillment of the common support condition.

According to the recommendations of Rosenbaum and Rubin (1985), the matching should reduce covariate imbalance for all variables. Table 4 illustrates that, before matching, covariates such as gender, family background, and majors significantly differed between the treated and control groups, with bias ranging from 13.4% to 15.2%. Furthermore, the *t*-test also showed significant differences. On the contrary, after matching, the means and variances of all covariates were consistent, with a bias approaching zero, and the *t*-test indicated no significant difference between the treated and control groups. Hence, after the matching process, students are balanced in both groups.

### 5.4. Propensity Score Matching Results

Table 5 displays the ATT of receiving VIVE on individuals’ vocational identity, self-efficacy, and job satisfaction. It applied different numbers of nearest neighbors, selected taking the sample size into account, and different caliper widths, often ranging from 0.01 to 0.05 (Austin, 2011), to ensure balance between the treated and control groups. The data suggested that the ATT of receiving VIVE had minimal and statistically negligible effects on individuals’ vocational identity, self-efficacy, and job satisfaction. Students with LDVE showed no significant changes in vocational identity, self-efficacy, or job satisfaction compared to their counterparts. Therefore, hypotheses H1, H2, and H3 were invalid.

### 5.5. Robustness Analysis

In this study, the robustness of the estimation results was mainly verified by replacing the PSM method and changing the matching method. Common PSM methods include nearest neighbor matching, kernel matching, Mahalanobis distance matching, and caliper and radius matching. Consistent results among different matching methods can indicate the robustness of the matching results (Chen, 2014). Therefore, different matching methods were used to guarantee the reliability of the estimation results. Table 5 demonstrates that the results of five estimation methods were consistent, with none suggesting that participation in VIVE significantly influences individuals’ vocational identity, self-efficacy, or job satisfaction.

The primary concern with PSM analysis is the issue of hidden bias due to selection on unobserved heterogeneity. Usually, the unbalanced effects of hidden bias can be eliminated through the bias correction method. Therefore, to validate the robustness of the estimation results, the ATT was re-evaluated using the Inverse Probability Weighted Matching (IPWM), which is more sensitive to hidden bias (C. Shen et al., 2011; Allan et al., 2020). The results showed that the ATT of participation in VIVE programs on individuals’ vocational identity, self-efficacy, and job satisfaction were all small, with *p*-values exceeding 0.05, indicating that the effects were statistically insignificant using the IPWM method and that the study’s results were robust (see Table 6).

### 5.6. Heterogeneity Analysis

To further understand the ATT of VIVE programs, the study employed the K-nearest neighbor matching method with a caliper (caliper = 0.01, k = 3) to analyze the effects of different models on vocational identity, self-efficacy, and job satisfaction. The estimation results are shown in Table 7. The findings indicated that LDVE had no significant impact on an individual’s vocational identity, self-efficacy, and job satisfaction, neither the secondary-to-higher nor the higher-to-undergraduate vocational education programs.

## 6. Conclusions and Discussions

### 6.1. Conclusions

Numerous studies have analyzed the significant positive impacts of vocational education on vocational identity, self-efficacy, and job satisfaction. However, many studies have overlooked the issue of sample selection bias, which can lead to estimation inaccuracies. Furthermore, research on the effect of LDVE is relatively rare. This paper empirically examined the effect of LDVE on an individual’s vocational identity, self-efficacy, and job satisfaction using the PSM method. Three conclusions were ultimately drawn regarding the formulated hypotheses. First, LDVE exerted no significant impact on individuals’ vocational identity, thus failing to support Hypothesis 1. Second, LDVE showed no significant effect on individuals’ self-efficacy, indicating that Hypothesis 2 was invalid. Third, LDVE did not have a significant influence on individuals’ job satisfaction, which meant Hypothesis 3 was not supported.

Research has also found that family background and major would affect an individual’s likelihood of receiving LDVE. Furthermore, neither the secondary-to-higher nor the higher-to-undergraduate VIVE programs significantly impacted individuals’ vocational identity, self-efficacy, and job satisfaction.

In summary, merely extending the duration of vocational education is far from an effective strategy to enhance the positive impacts of vocational education. These findings offer a plausible explanation for the long-standing issue of skilled worker shortages. Specifically, our research failed to identify significant effects of long-duration vocational education (LDVE) on participants’ vocational identity, self-efficacy, or job satisfaction—factors that are widely recognized as critical to attracting more potential individuals to skilled occupations.

### 6.2. Discussion and Suggestions

The findings on the effects of family background and academic major on receiving LDVE are largely in line with previous studies on the impacts of individual, family, and learning factors on schooling. Although previous studies have found that gender influences an individual’s participation in education, these findings have been most evident in technical and engineering fields (Makarova et al., 2019; Lewis et al., 2021). This research has collected data from the business and nursing disciplines, which could attract many female students and yield an insignificant gender effect.

Evidence can also be found in existing research regarding the insignificant effect of LDVE on vocational identity, self-efficacy, and job satisfaction. Some studies have suggested that the marginal benefits of vocational education are more significant in the early stages of people’s careers; however, the marginal effects of vocational education may diminish as careers advance (Hanushek et al., 2017). Moreover, Blossfeld et al. (2014) noted that if individuals cannot adapt to rapidly changing work environments through further education and skills updates in their careers, the long-term benefits of vocational education will gradually diminish. Therefore, the superposition of marginal effects of vocational education at different stages may lead to insignificant overall impacts.

Forster et al. (2016) found that although vocational education can help individuals enter the job market more quickly at an early stage, its benefits may diminish over time due to insufficient skill adaptability. Based on these findings, repeated and outdated educational content in the VIVE program may lead to insignificant effects. Currently, VIVE in China usually involves the participation of two vocational schools. However, some studies have found that vocational schools at the higher level do not actively adjust their curriculum due to the ‘superiority’ of their academic system, resulting in the meaningless repetition of content, which fails to truly achieve vertically integrated talent cultivation (Lin & Wang, 2016; Ke & Shi, 2018; Nian, 2020).

Furthermore, several studies have identified instances where teachers exhibited academic prejudices against vocational students, perceiving them as less capable, less committed to learning, and lacking appropriate knowledge (H. C. Shen & Liu, 2020; Billett, 2014). This is performed by deliberately reducing the difficulty of the course and repeatedly saying that the courses they teach are simple. Such stereotypes about vocational students may also result in insignificant educational effects by providing poor instructional quality or adopting unfavorable pedagogical strategies.

Additionally, studies have demonstrated that students’ interests can influence their academic achievements (Chen et al., 2023). Currently, in China, there is a lack of comprehensive vocational guidance and advice on major selection before students receive LDVE. This may also lead students to enter a professional field that they did not initially intend to study. As a result, even if the opportunity for a VIVE program is provided, it may not be possible to effectively enhance their vocational identity, self-efficacy, and job satisfaction.

Regarding the external cause, fast and frequent occupational transitions may affect the impact of LDVE on vocational identity, self-efficacy, and job satisfaction. On one hand, a deep understanding and mastery of a specific skill or knowledge domain often require long-term study, practice, and accumulated experience. Frequent job changes can disrupt this process and potentially weaken one’s sense of vocational identity, self-efficacy, and job satisfaction (Lee & Mitchell, 2011). On the other hand, existing research has confirmed that the alignment between educational content, such as major design, and occupational demands is positively correlated with individuals’ vocational identity, self-efficacy, and job satisfaction (Wessel et al., 2008). The greater the alignment between the provided vocational education content and occupational demands, the higher the levels of vocational identity, self-efficacy, and job satisfaction individuals are likely to achieve. However, nowadays, individuals face more frequent job changes than in the past, which may result in a mismatch between vocational education content and occupation requirements, thereby reducing the effectiveness of vocational education in enhancing vocational identity, self-efficacy, and job satisfaction.

Based on the potential reasons for the insignificant effects outlined above, this study proposes the following recommendations for relevant stakeholders, including the government, educational institutions, and industries. First, it is advised that educational policymakers and regulatory bodies implement multiple strategies to effectively attract skilled talent. The study findings indicate that merely extending the duration of vocational education does not significantly improve vocational identity, self-efficacy, and job satisfaction. Recommended strategies are better supplemented with structured academic and career guidance systems and high-quality LDVE programs, which consider the socioeconomic backgrounds and aptitude for specific vocational disciplines of potential skilled talents. It is suggested that prior to enrolling in LDVE programs, relevant consultations and assessments regarding majors and personality traits should be provided, along with career counseling for future employment. Such an approach may help learners select majors that align with their individual characteristics, thereby contributing to the realization of the positive effects of long-duration vocational education.

Second, it is recommended that vocational schools involved in VIVE programs proactively and equally contribute to the formulation of student development plans and cooperate in delivering progressive educational content as the level of education rises, attempting to exert positive marginal effects of LDVE. Furthermore, based on the good cooperation among vocational educational institutions, it is suggested that the quality of educational content be improved. On the one hand, the content of vocational education at different educational stages must demonstrate differentiation and progression to ensure continuous knowledge updates across all educational stages. On the other hand, the content of vocational education at each stage must be consistently in line with external demand to avoid obsolescence and regression. It is suggested that partner schools engaged in long-duration vocational education should establish a regular cooperation mechanism and share relevant instructional resources and strategies. This will enable them to provide learners with coherent and progressive learning content, thereby avoiding redundant or disjointed learning experiences in LDVE programs.

Third, industry specialists and experts are encouraged to participate in designing and developing vocational curricula to ensure that vocational educational content is regularly updated to reflect the latest developments in industry and technology. In particular, driven by evolving industry demands, the rise in emerging professions, and continuous technological advancements, the curricular restructuring prioritizes cultivating a diverse skill set essential for students to confront the fast and frequent transition in occupations. Meanwhile, it suggests establishing a systematic feedback mechanism to collect evaluations from students, educators, parents, company managers, and industry experts regarding the educational content, thus ensuring that the instruction keeps pace with industry and technological developments.

### 6.3. Shortcomings and Prospects

This study investigated the impacts of LDVE on the vocational identity, self-efficacy, and job satisfaction of Chinese vocational students. It applied convenience sampling in this research, but given the heterogeneity of vocational education systems across countries, the generalizability of this study requires further investigation in the future using a wider range of data from other countries and various sampling methods. Taking the limitations of the PSM method into account, although some tests have been conducted to verify the results’ robustness, the results’ accuracy could be further improved by using other statistical analytic methods. To better support the findings of this study, future research could examine other models, such as those that consider the interaction effects between variables. As this study is at the preliminary exploratory stage regarding the impact of LDVE, and given the time and funding constraints, no formal sensitivity analyses were conducted. Therefore, it is advised that future studies include relevant sensitivity analyses to validate this study’s findings further. Additionally, despite proposing several potential explanations for the insignificant impacts, further verification is essential in future research.

## Figures and Tables

**Table 1:** Description of the respondents (n = 1878).

Variable	Category	n	Percentage (%)
Gender	Male	867	46.2
	Female	1011	53.8
Family Background	Urban	761	40.5
	Rural	1117	59.5
Educational Level	Secondary vocational school	387	20.6
	Vocational college	1125	59.9
	Vocational university	366	19.5
Major	Engineering and manufacturing	503	26.8
	Business	704	37.5
	Medicine and nursing	314	16.7
	IT	357	19.0

**Table 2:** Estimation before matching.

Variable	Treated		Control		*t*	*p*
	M	SD	M	SD		
Vocational identity	32.04	8.21	31.80	8.010	0.44	0.66
Self-efficacy	32.73	7.76	32.83	7.515	0.49	0.62
Job satisfaction	27.21	6.93	27.03	6.789	−0.20	0.84

**Table 3:** Estimates of individuals receiving VIVE.

Variable	β	SE	Odds Ratio
Constant	−0.35 *	0.16	0.61
Gender	−0.13	0.69	0.80
Family Background	−0.16 *	0.69	0.75
Major	−0.07 *	0.03	0.88
Log likelihood	−857.79		
Pseudo R^2^	0.01		
LR Chi^2^	14.81		

Note: * *p* < 0.05.

**Table 4:** Covariables after PSMS.

Variable	Matching	Mean		SE	ASMD	% Bias	*t*-Test	
		Treated	Control				*t*	*p* > |*t*|
Gender	Unmatched	1.48	1.55	−13.4	0.134	100	−2.2	0.028
	Matched	1.48	1.48	0	0		0	1.000
Family Background	Unmatched	1.54	1.61	−14.5	0.145	100	−2.4	0.016
	Matched	1.54	1.54	0	0		0	1.000
Major	Unmatched	2.15	2.31	−15.2	0.152	100	−2.48	0.013
	Matched	2.15	2.15	0	0		0	1.000

**Table 5:** Average treatment effect estimation with different PSM methods.

Variable	Matching Method	Treated	Control	ATT (%)	*t*
Vocational identity	K-NN matching with caliper (k = 3, caliper = 0.01)	32.037	33.304	−1.267	0.82
	K-NN matching (k = 2)	32.037	32.230	−0.193	0.29
	Kernal matching	32.037	31.718	0.319	0.64
	MD matching	32.037	31.787	0.249	0.14
	Caliper matching (caliper = 0.05)	32.037	32.158	−0.121	0.21
	Mean	32.037	32.239	−0.203	-
Self-efficacy	K-NN matching with caliper (k = 3, caliper = 0.01)	32.732	33.216	−0.484	0.34
	K-NN matching (k = 2)	32.732	32.998	−0.266	0.43
	Kernal matching	32.732	32.761	−0.028	0.06
	MD matching	32.732	32.563	0.169	0.10
	Caliper matching (caliper = 0.05)	32.732	33.232	−0.500	0.94
	Mean	32.732	32.954	−0.222	-
Job satisfaction	K-NN matching with caliper (k = 3, caliper = 0.01)	27.212	28.234	−1.022	0.84
	K-NN matching (k = 2)	27.212	27.333	−0.121	0.22
	Kernal matching	27.212	26.959	0.252	0.60
	MD matching	27.212	27.628	−0.415	0.34
	Caliper matching (caliper = 0.05)	27.212	27.296	−0.084	0.18
	Mean	27.212	27.49	−0.278	-

Note: NN nearest neighbor, MD Mahalanobis distance.

**Table 6:** Average treatment effect estimation with bias correction.

Variable	ATT (%)	SE	Z-Value	*p*
Vocational Identity	0.266	0.517	0.52	0.606
Self-efficacy	−0.133	0.489	−0.27	0.785
Job Satisfaction	0.172	0.441	0.39	0.696

**Table 7:** Average treatment effect estimation of different types of VIVE programs.

Variable	Integration Type	n	Treated	Control	ATT (%)	*t*
Vocational identity	secondary-to-higher	178	32.46	32.12	0.39	0.54
	higher-to-undergraduate	147	31.53	31.73	−0.209	−0.27
Self-efficacy	secondary-to-higher	178	32.9878	33.03	−0.05	−0.08
	higher-to-undergraduate	147	32.44	32.73	−0.29	−0.41
Job satisfaction	secondary-to-higher	178	27.58	27.29	0.29	0.53
	higher-to-undergraduate	147	26.77	27.00	0.23	−0.37

## Data Availability

The data are available upon request.

## Abbreviations

The following abbreviations are used in this manuscript:

LDVE	Continuous and prolonged vocational education
VIVE	Vertically integrated vocational education
PSM	Propensity score matching
ATT	Average treatment effect of the treated
